# Too Little of a Good Thing

**Published:** 1885-10

**Authors:** J. G. Templeton

**Affiliations:** Pittsburgh


### TOO LITTLE OF A GOOD THING.

BY J. G. TEMPLETON, D. D. S., PITTSBURGH.

READ BEFORE THE PENNSYLVANIA STATE SOCIETY AT ITS SEVENTEENTH ANNUAL MEETING AT CRESSON.

"A little learning is a dangerous thing,
Drink deep, or taste not the Pierian spring,
For shallow draughts intoxicate the brain,
But drinking largely sobers us again."

It is not our purpose to give even an epitomized history of education. Neither do we intend to specify minutely all the particular studies that should form a part or all of the preliminary training of the dental student.

But as we begin to write, the thoughts of what is needed come thick and fast. Swift memory reverts to student days and college associations as the most pleasant of life.

Though we write in waking hours, we cannot refrain from quoting those beautiful lines of the poet, Thos. Moore, where, in speaking of his memories, he says:

> "Oft in the stilly night,
> Ere slumber's chain has bound me,
> Fond memory brings the light
> Of other days around me."

This same memory would be treacherous indeed if it could not, with college life, recollect some things in connection with Grecian and Roman history.

We wish to apply to preliminary education for the dental student what Demosthenes said when he was asked "What is the true relation of Athens to Greece?" His reply was, "Athens is the natural head of Greece." Such, in his belief, was the part that Athens must perform if Greece was to be safe and progress as a nation.

As Athens was to Greece, so stands a thorough education in its relation to progress in our profession. The evils to be cured are but different phases of one malady. To accomplish our purpose this education must be such as to fully develop the intellectual faculties.

It must not be one-sided, like the training which prevailed at one time in that great empire that ruled the world from the city of the Seven Hills. The Romans understood no system of training but that of oratory. They knew no science of government or of political economy. Cicero, the great Roman orator, who could fire the Roman Senate with his eloquence, and excite the Roman people to undertake gigantic wars and gain mighty conquests, speaks lightly of jurisprudence. "Any one" says he, "can make himself a jurisconsult in a week, but an orator is the production of a lifetime." In his day an orator was thought to be a perfect man, but this barrier was broken by or through the influence of Quintilian, who gave a system of training, though intended only for public speakers, but of which Milton says it would "fit a man to perform justly, wisely, and magnanimously all the offices, both public and private, of peace and war." If, in the opinion of such a man as Milton, such grand results could be obtained from the teachings of one who lived in the time of Emperors Vespasian and Domitian, eighteen centuries ago, where is our boasted progress; or do we ask too much if we demand that a student of dentistry shall have such an education as will fit him to perform any or all the duties of any office in the gift of his fellow-citizens?

The satisfaction, the pleasure derived, and the benefit to any person having a liberal education, amply repay its possessor for all the time, study, or labor and money spent in obtaining it; in brief, it is worth all it costs.

The aim of all should be a good preliminary training. The time thus spent is time gained afterward in the facility of comprehending whatever is brought before the mind. This mental discipline of which we speak, and which we deem so important and necessary, is the sure foundation for the superstructure of scientific professional knowledge. With the understanding of the origin and derivation of words comes their meaning, and the dictionary is not so often needed. The educated mind sees and immediately admires the grand beauty of the comprehensive technical term, which is not only a name, but also a definition as well. With qualifications as above indicated, progress in professional knowledge may be rapid and comparatively easy, according to the amount of energy and persistence of the student.

Preparatory work does not come within the province of professional schools of any kind.

In all our dental and medical colleges the curriculum of study is based on the supposition that all who come to these institutions have had the advantages of a liberal, or at least a sufficient preliminary education, that will qualify the student to receive the lectures intelligently, with a readiness of perception and retentive memory, which, with rare exception, is only acquired by a proper course of mental discipline; hence the educated student at the dental college receives more for his time and money spent in attendance at the lectures, because of his greater mental capacity. From his familiarity with the original languages, he understands all nomenclature, and sees a beauty in the adaptation which the scholar only can see. Besides his having a knowledge of the natural sciences, he is prepared to grasp their application to professional subjects the moment they are announced by the lecturer. With such advantages in favor of preliminary attainments, why should we not as a profession demand it of our students? Some one says he is too poor; he cannot afford the expense connected therewith. All we have to say to such objectors is that "Where there is a will there is a way." The avenues to learning are not closed, and all who desire can avail themselves of the advantages offered in the literary institutions of our country. Here it is worthy of remark that very many of the best and most prominent men of all professions are those who have earned the money with which to educate themselves.

There are many points incidental to our theme that might be elaborated, but the limits of this paper forbid, and I forbear, with one or two exceptions.

We look with pride and satisfaction on the reputation that American dentistry has attained abroad for progress and perfection. We have heard how the phrase "American Dentist," displayed on signs, has been the passport to many a lucrative practice. Yet none know better than we do of the vast amount of suffering, deformity, and disfigurement occasioned by the ignorance of so-called dentists in this country. Such things occur all around us, every-day, at the hands of men who know too little to know that they know but little.

That this elaborate training of which we have spoken is needed in dental education cannot be successfully controverted; then why should we not, as a profession, use every effort possible to close all short roads to dental practice, and impress upon the minds of all the necessity of learning also that:—

> "Learning by study must be won;
> 'Twas ne'er entailed from sire to son."

